# Supplementary material for: The Efficacy and Safety of Immune Checkpoint Inhibitors in Adrenocortical Carcinoma: A Systematic Review and Meta-Analysis
Source: Cancers (Basel). 2024 Feb 23;16(5):900. doi: 10.3390/cancers16050900 (PMC10931182; doi:10.3390/cancers16050900)
Supplement: Supplementary file 1 [file cancers-16-00900-s001.zip › cancers-2839751-supplementary.pdf]

Supplementary material

Figure S1. ORR based on the used treatment.

Figure S2. DCR based on the used treatment.

Figure S3. 6-months overall survival.

Figure S4. 6-months progression-free survival.

Figure S5. Grade III/IV adverse events.

Table S1. Search algorithm.

Table S2. Quality appraisal of included studies according to MINORS assessment tool.

Table S3. Quality appraisal of included Case Reports studies according to JBI Critical Appraisal Checklist

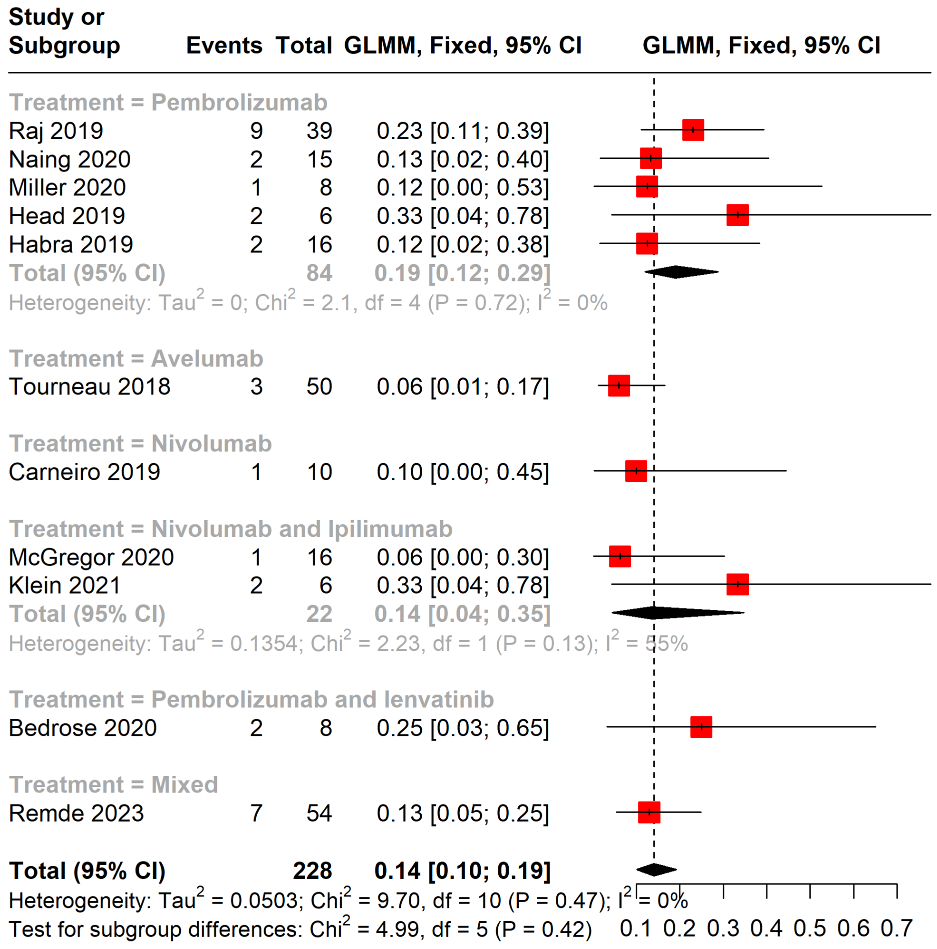

Figure S1. ORR based on the used treatment [16,17,21,23–25,29–32].

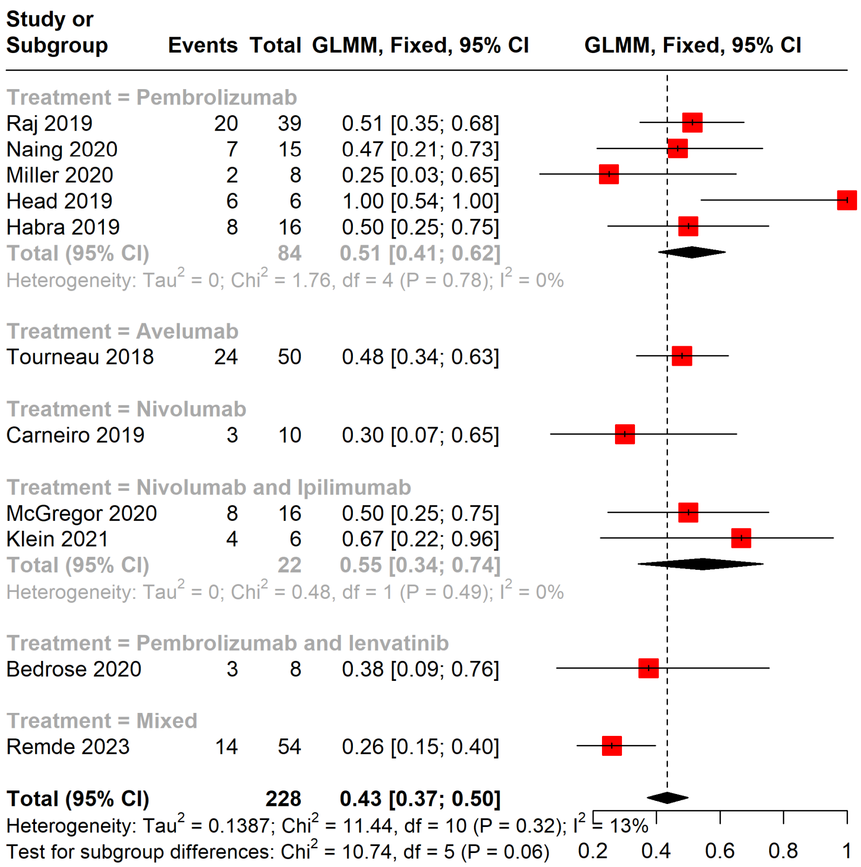

Figure S2. DCR based on the used treatment [16,17,21,23–25,29–32].

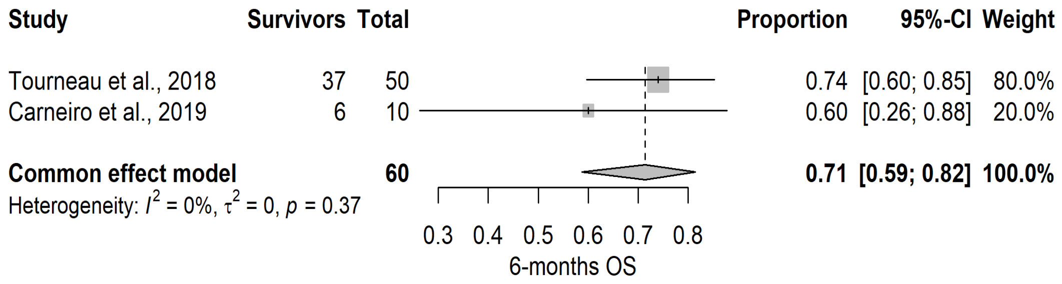

Figure S3. 6-months overall survival [17,23].

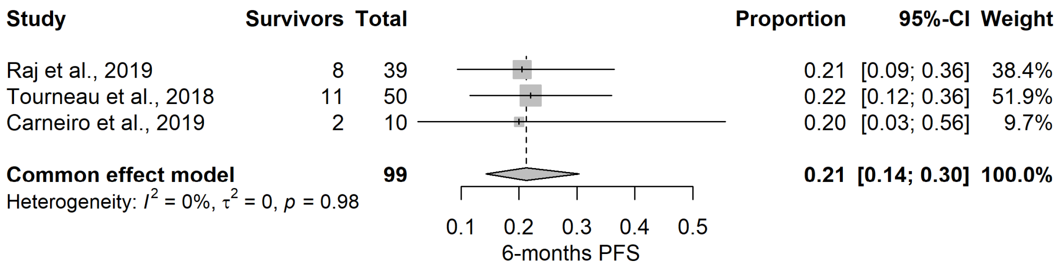

Figure S4. 6-months progression-free survival [16,17,23].

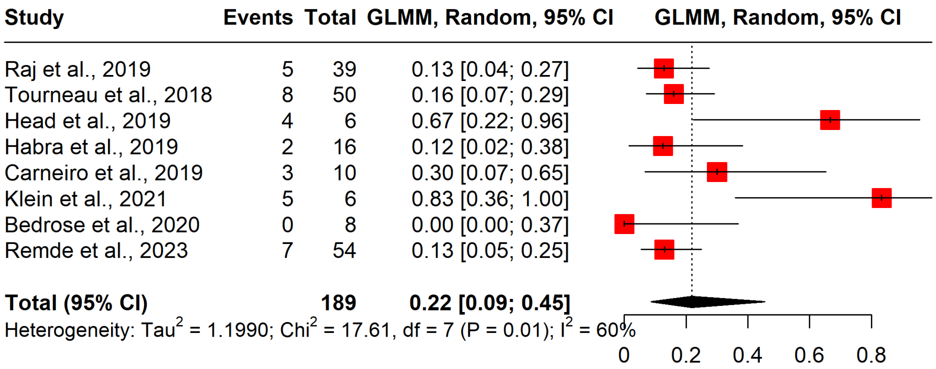

Figure S5. Grade III/IV adverse events [16,17,23,25,30–32].

Table S1. Search algorithms.

PubMed
(((adrenocortical[Title/Abstract]) OR (acc[Title/Abstract])) OR (adrenal*[Title/Abstract])) AND ((((((((((immune checkpoint inhibitors[Title/Abstract]) OR (ipilimumab[Title/Abstract])) OR (nivolumab[Title/Abstract])) OR (pembrolizumab[Title/Abstract])) OR (avelumab[Title/Abstract])) OR (cemiplimab[Title/Abstract])) OR (tremelimumab[Title/Abstract])) OR (durvalumab[Title/Abstract])) OR (atezolizumab[Title/Abstract])) OR (spartalizumab[Title/Abstract])) OR (immunotherapy[Title/Abstract]))
Scopus
((TITLE-ABS-KEY (adrenocortical)) OR (TITLE-ABS-KEY (acc)) OR (TITLE-ABS-KEY (adrenal*))) AND ((TITLE-ABS-KEY ("immune checkpoint inhibitors")) OR (TITLE-ABS-KEY ("ipilimumab")) OR (TITLE-ABS-KEY ("nivolumab")) OR (TITLE-ABS-KEY ("pembrolizumab")) OR (TITLE-ABS-KEY ("avelumab")) OR (TITLE-ABS-KEY ("cemiplimab")) OR (TITLE-ABS-KEY ("tremelimumab")) OR (TITLE-ABS-KEY ("durvalumab")) OR (TITLE-ABS-KEY ("atezolizumab")) OR (TITLE-ABS-KEY ("spartalizumab")))
CENTRAL
(Adrenocortical carcinoma)

Table S2. Quality appraisal of included studies according to MINORS assessment tool.

Study	A clearly stated aim	Inclusion of consecutive patients	Prospective collection of data	Endpoints appropriate to the aim of the study	Unbiased assessment of the study endpoint	Follow-up period appropriate to the aim of the study	Loss to follow up less than 5%	Prospective calculation of the study size	Overall Appraisal score
Remde2023	2	2	0	2	1	2	1	0	10
Edenfield2021	2	2	2	2	2	2	2	0	14
Klein2021	2	2	2	2	1	2	2	1	14
Bedrose2020	2	2	0	2	1	2	2	1	12
McGregor2020	2	2	2	2	1	2	1	2	14
Miller2020	2	2	0	2	1	2	2	0	11
Naing2020	2	2	2	2	1	2	1	2	14
Carneiro2019	2	2	2	2	2	1	1	2	14
Geoerger2019	2	2	2	2	1	1	2	2	14
Habra2019	2	2	2	2	1	2	1	1	13
Head2019	2	2	0	2	1	2	2	0	11
Raj2019	2	2	2	2	2	2	2	2	16
Tourneau2018	2	2	2	2	1	2	2	2	15
Sakamouri2017	2	2	2	2	1	2	2	0	13

Table S3. Quality appraisal of included Case Reports studies according to JBI Critical Appraisal Checklist.

1. Were patient's demographic characteristics clearly described?
2. Was the patient's history clearly described and presented as a timeline?
3. Was the current clinical condition of the patient on presentation clearly described?
4. Were diagnostic tests or assessment methods and the results clearly described?
5. Was the intervention(s) or treatment procedure(s) clearly described?
6. Was the post-intervention clinical condition clearly described?
7. Were adverse events (harms) or unanticipated events identified and described?
8. Does the case report provide takeaway lessons?

Study	Q1	Q2	Q3	Q4	Q5	Q6	Q7	Q8
Alam2021 [36]	YES	YES	YES	YES	YES	YES	NO	YES
Caccese2019 [35]	NOT CLEAR	YES	YES	YES	YES	YES	YES	YES
Casey2018 [34]	NOT CLEAR	YES	YES	YES	YES	YES	YES	YES
Mota2018 [33]	YES	YES	YES	YES	YES	YES	YES	YES
Weng2023 [37]	YES	YES	YES	YES	YES	YES	YES	YES
Charles2023 [38]	YES	YES	YES	YES	YES	YES	YES	YES
